# Visualizing Patient Pathways and Identifying Data Repositories in a UK Neurosciences Center: Exploratory Study

**DOI:** 10.2196/60017

**Published:** 2024-12-24

**Authors:** Jo Knight, Vishnu Vardhan Chandrabalan, Hedley C A Emsley

**Affiliations:** 1 Lancaster Medical School, Lancaster University, Bailrigg, Lancaster, LA1 4YW, United Kingdom, 44 01524 594547; 2 Lancashire Teaching Hospitals NHS Foundation Trust, Preston, United Kingdom

**Keywords:** health data, business process monitoring notation, neurology, process monitoring, patient pathway, clinical pathway, patient care, EHR, electronic health record, dataset, questionnaire, patient data, NHS, National Health Service

## Abstract

**Background:**

Health and clinical activity data are a vital resource for research, improving patient care and service efficiency. Health care data are inherently complex, and their acquisition, storage, retrieval, and subsequent analysis require a thorough understanding of the clinical pathways underpinning such data. Better use of health care data could lead to improvements in patient care and service delivery. However, this depends on the identification of relevant datasets.

**Objective:**

We aimed to demonstrate the application of business process modeling notation (BPMN) to represent clinical pathways at a UK neurosciences center and map the clinical activity to corresponding data flows into electronic health records and other nonstandard data repositories.

**Methods:**

We used BPMN to map and visualize a patient journey and the subsequent movement and storage of patient data. After identifying several datasets that were being held outside of the standard applications, we collected information about these datasets using a questionnaire.

**Results:**

We identified 13 standard applications where neurology clinical activity was captured as part of the patient’s electronic health record including applications and databases for managing referrals, outpatient activity, laboratory data, imaging data, and clinic letters. We also identified 22 distinct datasets not within standard applications that were created and managed within the neurosciences department, either by individuals or teams. These were being used to deliver direct patient care and included datasets for tracking patient blood results, recording home visits, and tracking triage status.

**Conclusions:**

Mapping patient data flows and repositories allowed us to identify areas wherein the current electronic health record does not fulfill the needs of day-to-day patient care. Data that are being stored outside of standard applications represent a potential duplication in the effort and risks being overlooked. Future work should identify unmet data needs to inform correct data capture and centralization within appropriate data architectures.

## Introduction

In the United Kingdom, the National Health Service (NHS) is the largest health care provider, and as such, it holds a large amount of complex and sensitive data. A recent study mapped NHS data flows at a national level, highlighting its scale and complexity, with a lack of transparency, duplication of datasets, and failure to follow best practices for data safety, as well as the need to ensure the capability for electronic health record (EHR) data extraction [1]. Key studies released in the last few years have identified that better use of routine NHS data could lead to improvements in patient care, service delivery, research, and innovation, but that the NHS faces a number of challenges, including a lack of suitable data infrastructure [2,3]. One proposal arising from these studies was the development of national and subnational secure data environments (SDEs). Ongoing development of SDEs will enable unified access to previously disparate datasets. However, the utility of SDEs relies on the data they contain, which is dependent upon the engagement of stakeholders and the availability of appropriate infrastructure [1].

In this study, we use business process modeling notation (BPMN) to map data flows and data repositories within neurology services at a regional neurosciences center. BPMN is a visual modeling language, primarily used in business analysis, which follows a standardized set of symbols and grammar for depicting workflows. There are two stages to BPMN: the creation of a visual workflow and the execution of that workflow as an automated process. In this study, we focused on the first stage of creating visual process diagrams using BPMN grammar to understand how patient data are used, stored, and disseminated. We aimed to highlight the complexity of these processes, to identify any data repositories that are kept outside of the centralized EHR and allow for future improvements in data capture by identifying where unnecessary effort is being made. The benefits of using BPMN over other flowchart systems include the fact that is it easily understandable, standardized, and flexible [4]. Within a health care setting, BPMN has been used to model clinical processes, patient trajectories, and hospital protocols, and there have also been attempts to create health care–specific extensions to the BPMN grammar [5].

## Methods

### Overview

The study was conducted at the Lancashire Teaching Hospitals NHS Foundation Trust, a large NHS Trust in North West England providing secondary and tertiary services to a population of 1.5 million in Lancashire and South Cumbria, including the regional neurosciences center.

### Staff Included and Data Storage

We formed a multidisciplinary team consisting of a lead clinician (HE–a consultant neurologist), a data scientist, and a patient pathway coordinator. The initial goal of this team was to identify patient pathways and the data flows and storage associated with them. Three potential pathways were explored, including an outpatient pathway with a patient referred on a standard NHS 18-week timeline, an outpatient pathway where a patient was referred directly to diagnostic scanning as the referring clinician needed to exclude cancer, and an inpatient pathway. Initial flowcharts were drafted by this team that allowed the identification of areas in which more information was required. These informal diagrams were then translated into BPMN diagrams using the formalized grammar and notation.

We then consulted with staff from different departments to identify the details of the patient pathway and the relevant data flows and storage. We identified three key areas of discussion—how data were received by the department, how the department used and stored data when treating patients, and how data were disseminated to other departments or to the patient or general practitioner. The departments consulted included neurology subspecialties such as neurophysiology; specialist nurse teams for motor neuron disease, Parkinson disease, and epilepsy; as well as departments outside of direct neurology care, including radiology and phlebotomy, as neurology patients are often sent to these services for diagnostic testing. Following on from these discussions, the BPMN diagrams were updated to include the detail of the movement of data to, from, and between departments.

In addition to identifying the movement of data relating to neurology patient care, we identified where this data were being stored. There are several standard applications used by the hospital, including an EHR, as well as specialized systems for the collection and dissemination of complex data such as imaging results. We tracked the usage of these applications in the BPMN diagrams, but we also collected information about data storage outside of these standard systems. We were able to identify several separate spreadsheet and text files and other electronic data that were being used to directly enable patient care.

The final step after completing the BPMN diagrams and identifying data repositories was to collect information on the data being stored outside of the standard applications. For this, we used a simple questionnaire to request information from departments on the location of the dataset, the individual data fields collected, the purpose of the data, and how and where it is stored (Multimedia Appendix 1).

### Ethical Considerations

This study did not access any individual patient data, and as such, ethical approval was not required. The study was undertaken under a Service Evaluation agreement with the Lancashire Teaching Hospitals NHS Foundation Trust (Service Evaluation Ref: 427).

## Results

**Figure 1.**
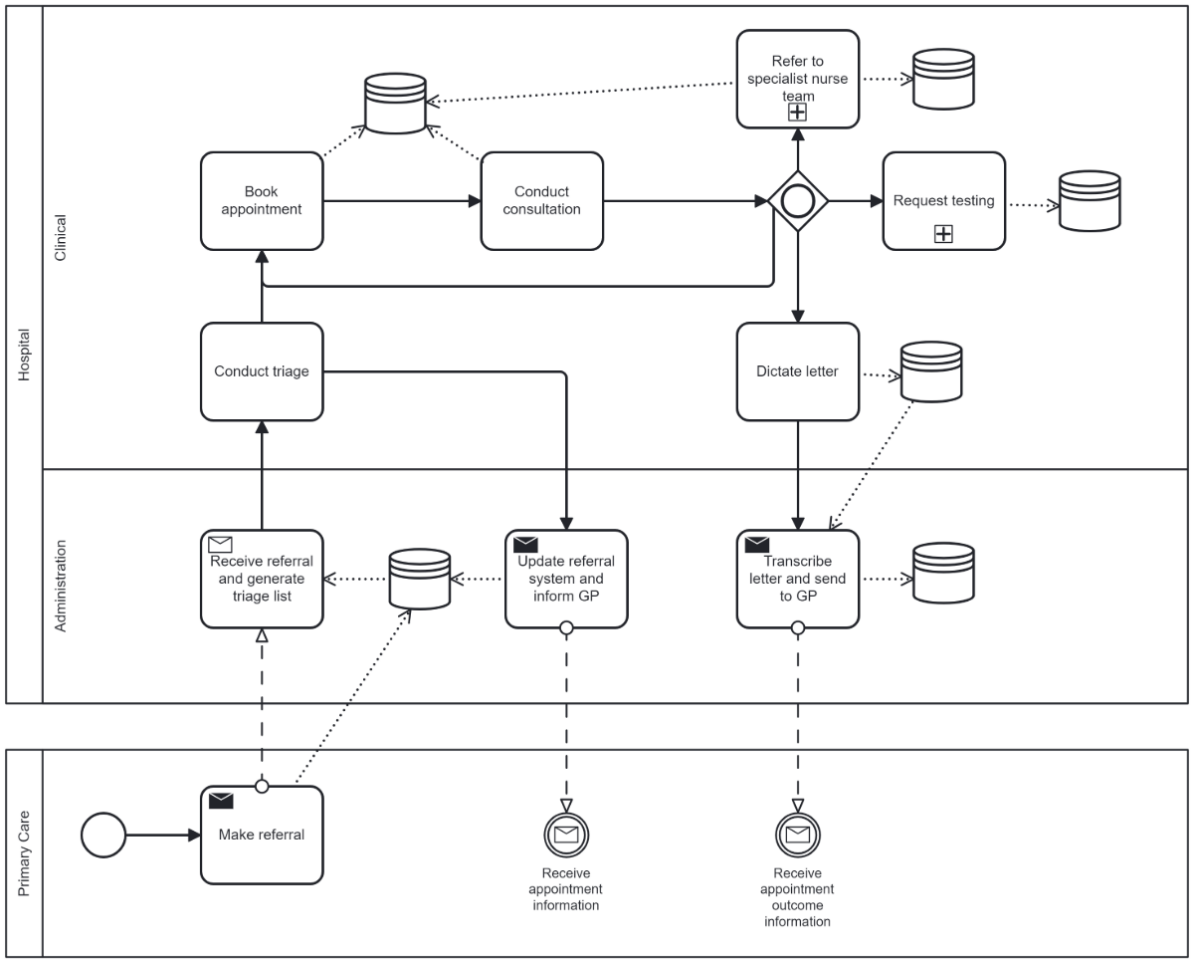
A simplified business process modeling notation diagram showing the patient journey through neurology services, and the corresponding points of data transfer and storage. GP: general practitioner.

BPMN was successfully used to visualize the patient pathway and the movement and storage of data. A simplified example of a diagram created for neurology outpatients can be seen in Figure 1. The original, more complex, diagrams can be found in Multimedia Appendix 2.

**Figure 2.**
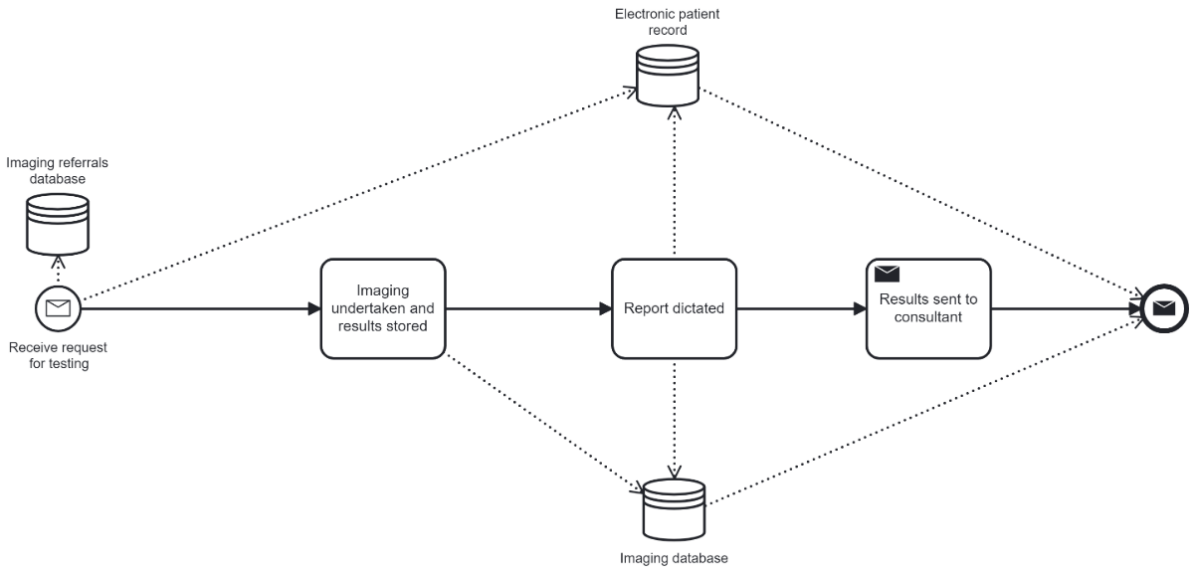
A business process modeling notation diagram showing a subprocess. This subprocess represents the flow of data to, through and from diagnostic imaging services.

A description of BPMN terminology and how to read the diagrams can be found in Textbox 1. We used a subprocess within the BPMN diagram to simplify the presentation, and these are denoted by a plus symbol within the task box. An example of the subprocess created for ordering tests from imagery is shown in Figure 2. For completeness, all process diagrams that were created as part of this project are available for viewing in the Multimedia Appendix 2.

Textbox 1. **A brief guide to business process modeling notation (BPMN) terminology.**

**BPMN symbols:**

- Rectangles represent tasks.
- Rectangles containing a plus sign represent subprocesses that contain their own process diagrams behind them (see subprocesses below).
- Cylinder icons represent data storage.
- Envelope icons represent messages and data being sent or received.
- Diamond icons represent decisions or ‘gateways.’

For more information on the BPMN protocols, please refer to the BPMN 2.0 symbol reference guide by Camunda [6].

**Subprocesses:**

Behind each subprocess is at least one separate process diagram. For example, the task box ‘request for testing’ has several subprocesses linked to it including separate process diagrams for requesting imaging, blood results, genetic testing, lumbar puncture, and so on.

**Reading the diagram:**

The process outlined in Figure 1 starts with a circle in the bottom left with the patient’s decision to seek health care. The process can then be followed by the direction of the arrows. There is a diamond shaped decision point following on from the task rectangle entitled “Conduct consultation”, and there are three possible outcomes of this decision – to refer to a specialist nurse, to request testing, or to dictate a letter. Both the task boxes for referral to a specialist nurse and requesting a test contain a plus sign, indicating that there is a separate subprocess behind each of these tasks. Once the arrows have been followed to their conclusion, the process ends in double walled circles at the bottom of the diagram indicating that information is provided back to Primary Care.

We identified 13 standard applications where neurology clinical activity was captured as part of the patient’s EHR, and these were backed by independent, vendor-provided databases with minimal integration with the core electronic patient record software. These included separate applications (and databases) for managing referrals, outpatient activity, laboratory data, imaging data, and other diagnostics, as well as for prescribing medicines from the pharmacy and obtaining clinic letters. We also identified 22 distinct datasets outside of standard applications created and managed within the neurosciences department, either by individuals or teams. The majority of these were stored as Microsoft Excel spreadsheets with a few Microsoft Word documents. There was no consistent or agreed structure to these datasets. They were being used by individuals or teams to enable them to deliver direct patient care, and include datasets for tracking patient blood results, recording which patients are enrolled in trials, recording home visits, monitoring patients with certain conditions or who are on particular medications, and tracking triage status. Table 1 shows the types of data categories contained in these datasets and the frequency with which those categories are stored. The table shows all data categories that appear in more than one dataset. Other categories that appear in only one dataset include patient telephone number, next of kin, triage decisions, and other miscellaneous information. Most of the datasets are stored as Microsoft Excel spreadsheets with 1 patient represented per line; however, some of the data are stored in other ways, for example in separate Microsoft Word files for each patient, or in Microsoft Excel but with a separate tab for each patient.

**Table 1.** The categories of data that were found in the datasets stored outside of standard applications.

Data category recorded	Number of datasets in which it appears
Patient name	22
NHS number	17
Medication	12
Diagnosis	11
Hospital number	9
Outcome of patient contact/appointment	8
Address and/or postal code	7
Date of patient contact/appointment	7
Responsible consultant(s)	7
Planned follow-up interval or date	7
Relevant medical history	5
Date of birth	5
Date of death	4
General practitioner	3
Sex	3
Referral to other services	3
Planned interval or date for testing	3
Test results	3
Date referral received	3
Intervention start and end dates	3
Date of diagnosis	3
Comorbidities	2
Patient referral source	2

## Discussion

### Previous Research

BPMN has been previously used to model clinical pathways, and we have used a similar approach in this study to model and understand the data flows and storage that arise alongside the patient pathway. One of the first appearances of BPMN related to health care in the literature was a 2008 conference paper demonstrating how to apply BPMN to a pathology process [7]. The authors conclude that using BPMN allowed easy communication of the process, early detection of errors, and could lead to future improvements in the process. After this early adoption, many other have gone on to apply BPMN to health care, for example, comparing BPMN with accepted standards for depicting hazardous drug workflows [8], using BPMN for modeling national health care guidelines [9], translating FHIR (Fast Healthcare Interoperability Resources) plans into BPMN diagrams [10], and modeling cross-sector health care quality indicators [11].

### Principal Findings

We used BPMN to visualize the patient pathway and identify data flows and repositories, highlighting the volume and complexity of the data generated by and used within neurology services. The findings of this study have shown that there are numerous datasets being stored outside of the hospital’s standard applications that raises concerns about the duplication of effort and the need to ensure that this data are being captured by central systems and therefore included in any data uploaded to a secure data environment. This study gives us an understanding of where datasets are stored, how the standard applications are being used, and an insight into why data may not always be stored in those systems.

We found that the further a patient is removed from inpatient services, the less well the EHR and other standard applications worked for recording their care. EHRs and related systems are often designed around inpatient episodes. However, outpatient care is structured differently, clinician interaction with the EHR is also time pressured, and the EHR does not necessarily do the things that are required for patient care, such as tracking which patients are still waiting for test results. This leads to individual clinicians creating their own records and spreadsheets, which are necessary for patient care, but are potentially duplicating data and are stored in decentralized repositories disconnected from the EHR. This is particularly noticeable within the teams who treat patients in the community, as they find it necessary to create their own data repositories for activities such as tracking home visits, as well as for equipment ordering.

The need to use datasets outside of the standard applications for data capture and patient tracking has arisen from the evolution of data practices from previous paper-based systems and the essential need to facilitate the delivery of clinical care. Often the EHR and other systems commissioned by the hospital do not work for all situations, and clinicians are left in a position where they need to implement their own system in order to provide the care the patient needs. To improve the design and architecture of future EHRs and to ensure the correct data are captured by SDEs, it is essential to understand why the EHRs are currently not providing the capability required, and what steps need to be taken to ensure that future platforms are fit for all these purposes. This study has taken the first step in this direction, but more work needs to be done to ensure that data systems are functional, and that data are being properly captured.

In this study, we found that data were being stored outside of the standard applications, but we were not able to explore or quantify how much data were being stored in this manner. It is possible that the data being held in spreadsheets and text files are a duplicate of data already held in the EHR, in which case this represents an inefficiency in the system. However, it is also possible that the data represent information that is not being captured in the EHR, in which case, it is important to capture this to ensure that all patient data are available for patient care and transfer to SDEs. If data are being captured in more than one place, this also raises questions about what happens if the data differ between different systems, for example, if a patient record in the EHR shows one date of birth and a record with a clinician spreadsheet shows a different date, which should be trusted? Having multiple sources of data storage increases the likelihood of there being such discrepancies.

With regard to the specific needs of the neurology department within this hospital, this study has highlighted areas where there are commonalities in processes within different parts of the neurology pathways. This knowledge could lead to improved understanding of where efficiencies can be achieved. For example, we noted that all the six specialist nurse teams have different ways of capturing data, but the way in which patient data are communicated to them could be standardized. This would allow for a more streamlined and efficient approach.

Although this study focuses on one specialty in one hospital, the work presented here may be of value to other departments and regions. We have demonstrated that BPMN can be of use in visualizing data pathways as well as clinical processes and, although the specific insights generated from this work may not be generalizable to other areas, the methodology is. It would be of great interest to extend this work into other specialties within the hospital to discover whether the same need to create bespoke datasets applies in other areas where patient care is removed from the inpatient setting. It would also be of value to extend this work to other hospitals and other geographical areas to see whether the insights remain the same, or whether other neurology departments face different data challenges.

Future research should focus on understanding why staff have developed datasets outside of the standard applications. It is essential to understand what functionality the current systems are not providing and how we can design better systems so that data are captured correctly. The failure to capture data correctly at local levels will only be magnified once data are drawn into national and subnational SDEs. Another key area for future research is to prioritize discovering whether the data kept outside of the standard applications duplicates data within the EHR, or whether these datasets represent a source of information that is not being officially recorded. Where data are being duplicated and the same information is being kept in several sources, it will be important to understand not only why it is being kept that way, but also whether the data differ, and if so, which source of data should be considered the ‘single source of truth.’

### Conclusion

We have used BPMN to visualize patient pathways and the data flow and storage that arise alongside it within a neurology department in a large NHS hospital. We have highlighted the complexity of data flow and the existence of data repositories outside of the standard applications. Explicitly identifying these datasets has allowed us to identify areas in which the current EHR is not fulfilling the needs of day-to-day patient care. Future work needs to be done to identify the specific data needs that are not being met to ensure that any future data architectures are able to meet this need and ensure that data are correctly captured and centralized. This is especially pertinent as the NHS moves toward the use of SDEs; in order for the SDEs to be of most value, we need to ensure that the correct data are being captured at a local level.

## Supplementary material

10.2196/60017 Multimedia Appendix 1 Format of the Questionnaire.

10.2196/60017 Multimedia Appendix 2 Original business process modeling notation diagrams.

## Abbreviations

BPMN: business process modeling notation
EHR: electronic health record
FHIR: Fast Healthcare Interoperability Resources
NHS: National Health Service
SDE: secure data environment
