# Supplementary material for: Visualizing Patient Pathways and Identifying Data Repositories in a UK Neurosciences Center: Exploratory Study
Source: JMIR Med Inform. 2024 Dec 24;12:e60017. doi: 10.2196/60017 (PMC11707554; doi:10.2196/60017)
Supplement: Multimedia Appendix 1 [file medinform-v12-e60017-s001.docx]

Appendix 1 - Questionnaire

Q1. Name of person completing form: Free Text

Q2. Department: Free Text

Q3. What is the name of the dataset/spreadsheet you are answering for?: Free Text

Q4. For what purpose is this data kept? (eg ensuring patient information is accessible, individual case load tracking, tracking test results, monitoring prescriptions): Free text

Q5. Who uses this data?: Free Text

Q6. How is this data stored?:

- As a spreadsheet in rows and columns, where all the information about one patient is kept in one row, and information about multiple patients is kept in the same sheet.
- As a spreadsheet with separate tabs for each patient.
- As separate word documents for each patient.
- Other. Please describe.

Q7. What are the names of all the separate pieces of information you collect in this dataset? (eg Hospital number, NHS number, diagnosis, appointment date etc): Free text

Q8. Where is this data stored?: Free text
