# Supplementary figures and images for: Visualizing Patient Pathways and Identifying Data Repositories in a UK Neurosciences Center: Exploratory Study
Source: JMIR Med Inform. 2024 Dec 24;12:e60017. doi: 10.2196/60017 (PMC11707554; doi:10.2196/60017)

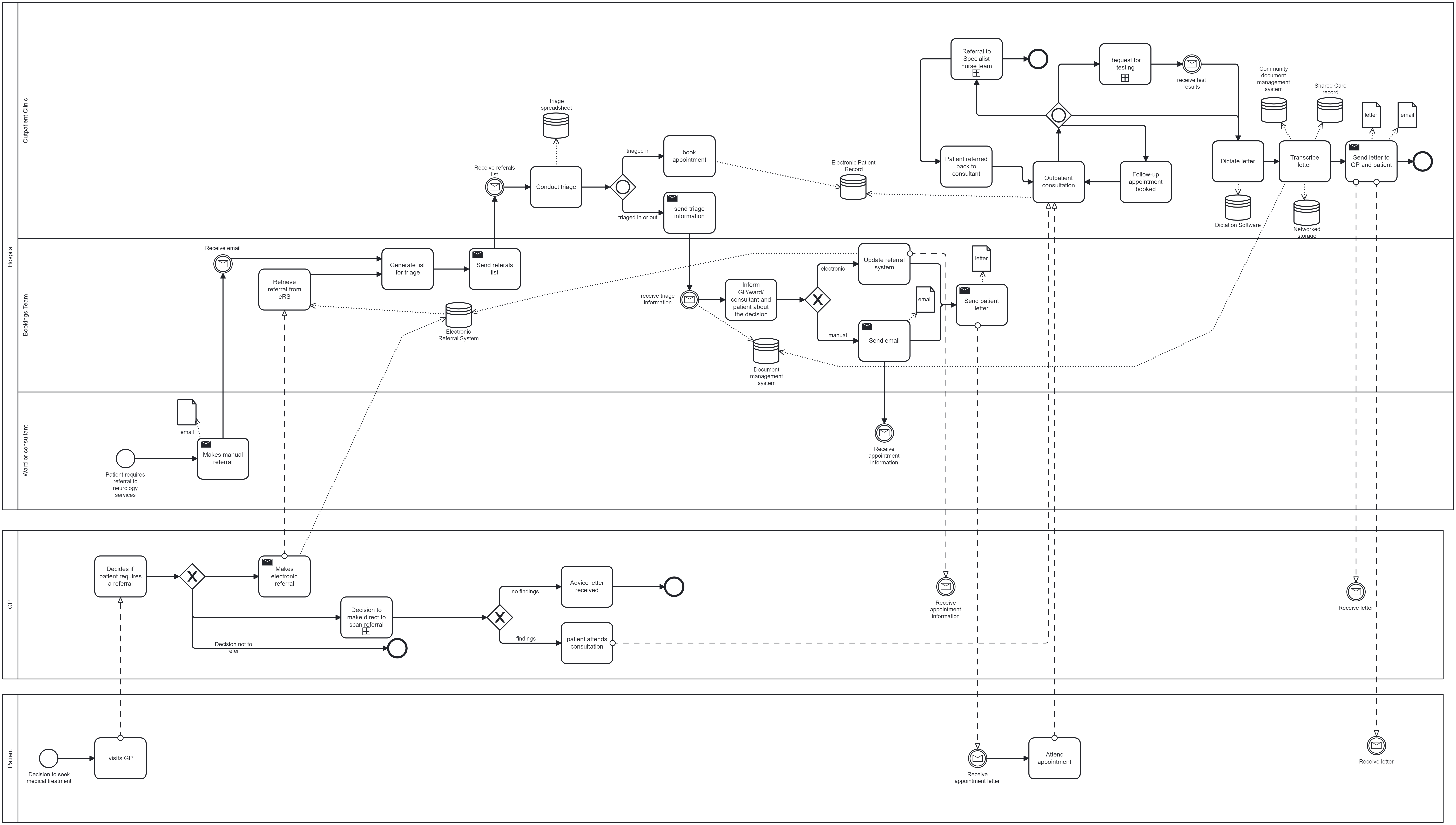

Supplement: Multimedia Appendix 2 [file medinform-v12-e60017-s002.zip › 60017-910928-1-SP.png]

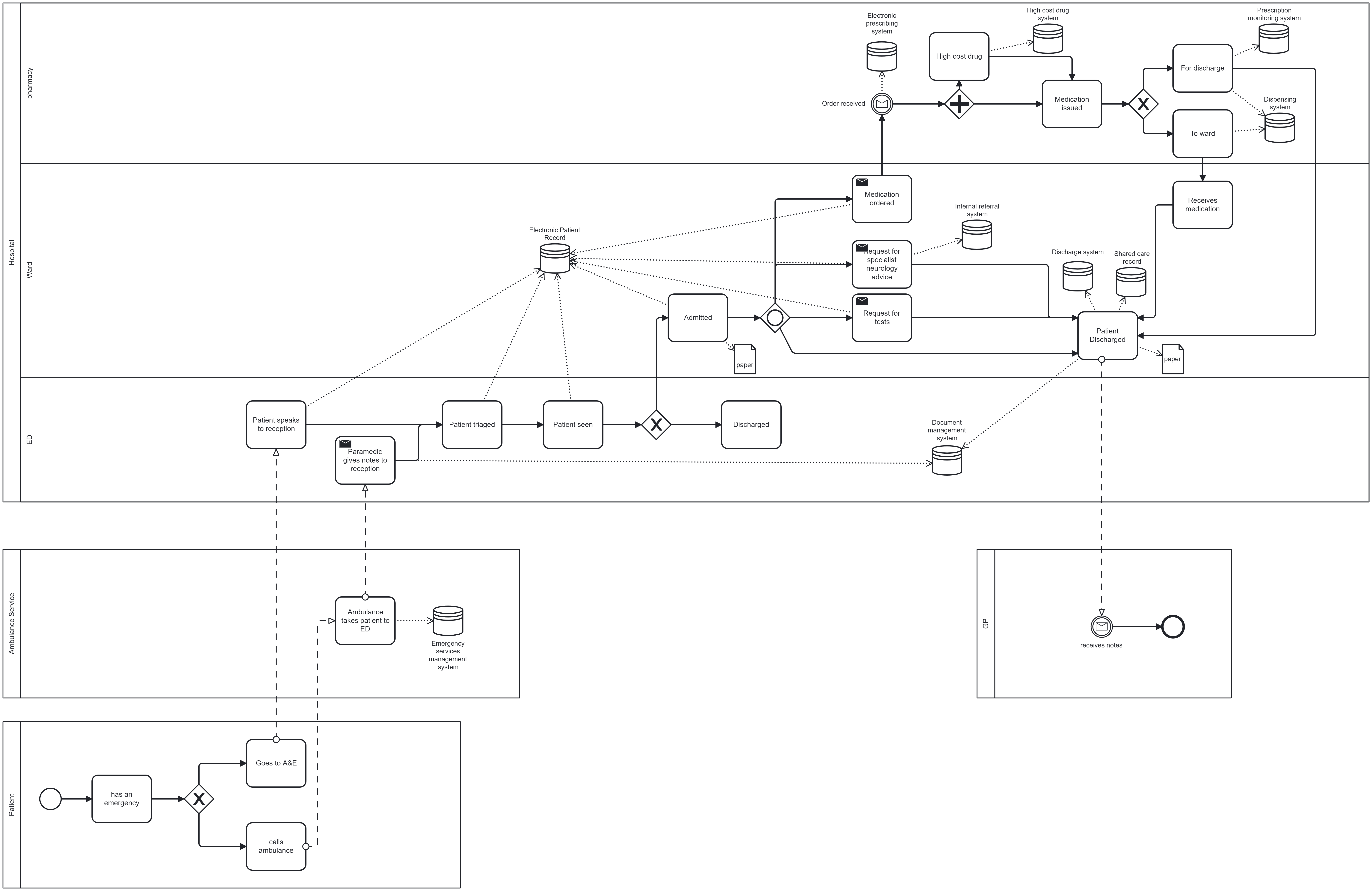

Supplement: Multimedia Appendix 2 [file medinform-v12-e60017-s002.zip › 60017-910931-1-SP.png]

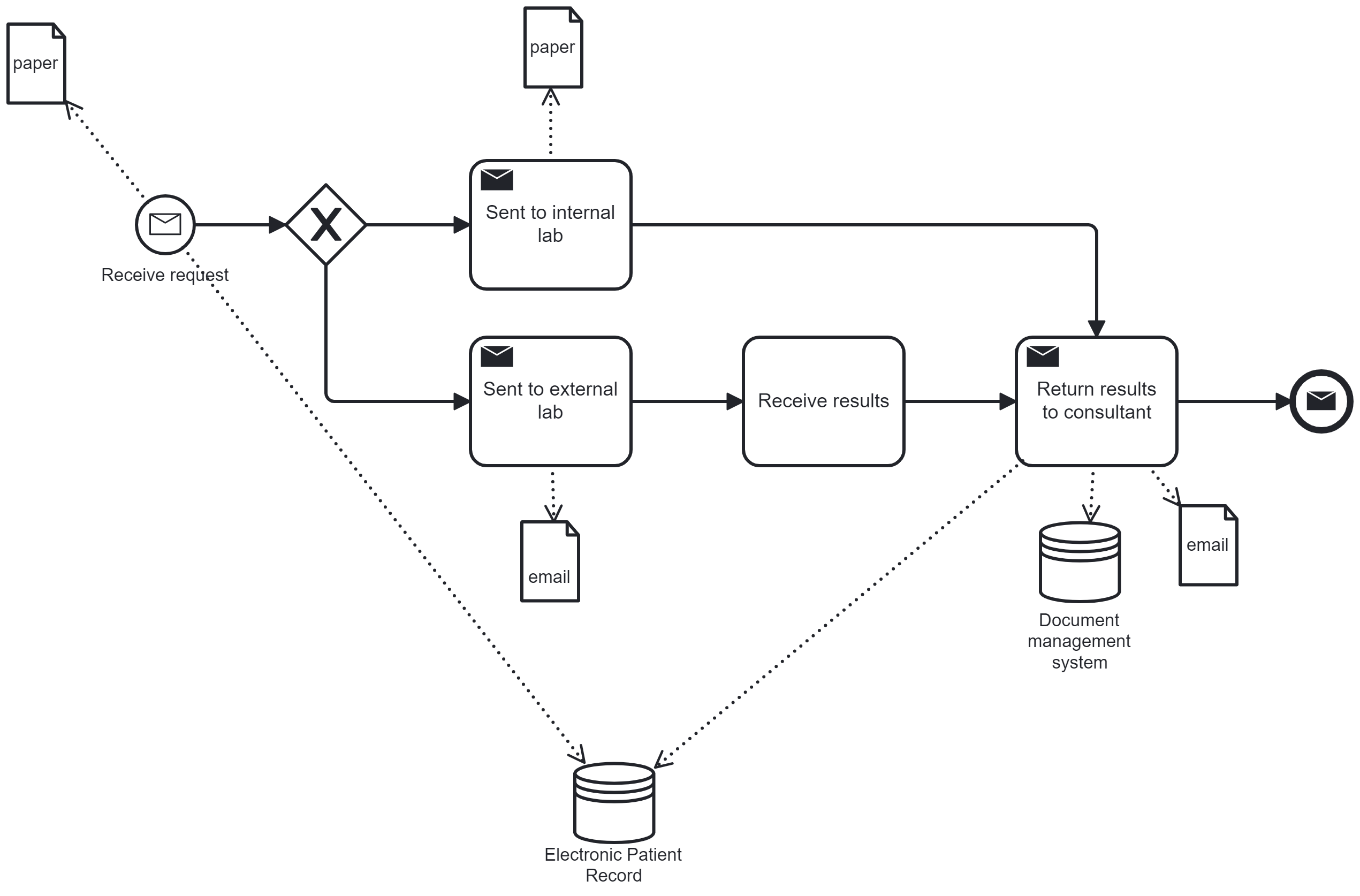

Supplement: Multimedia Appendix 2 [file medinform-v12-e60017-s002.zip › 60017-910932-1-SP.png]

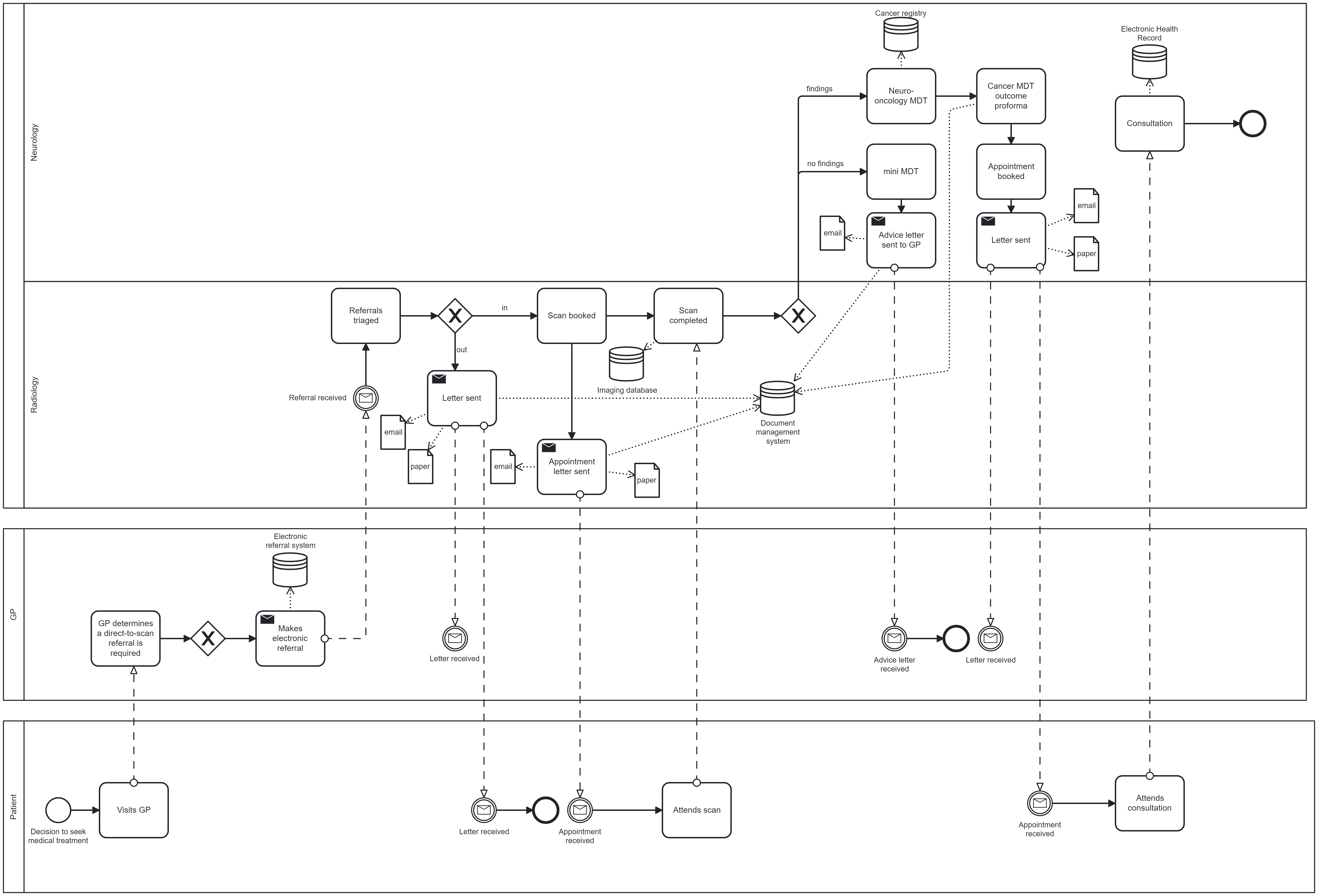

Supplement: Multimedia Appendix 2 [file medinform-v12-e60017-s002.zip › 60017-910934-1-SP.png]

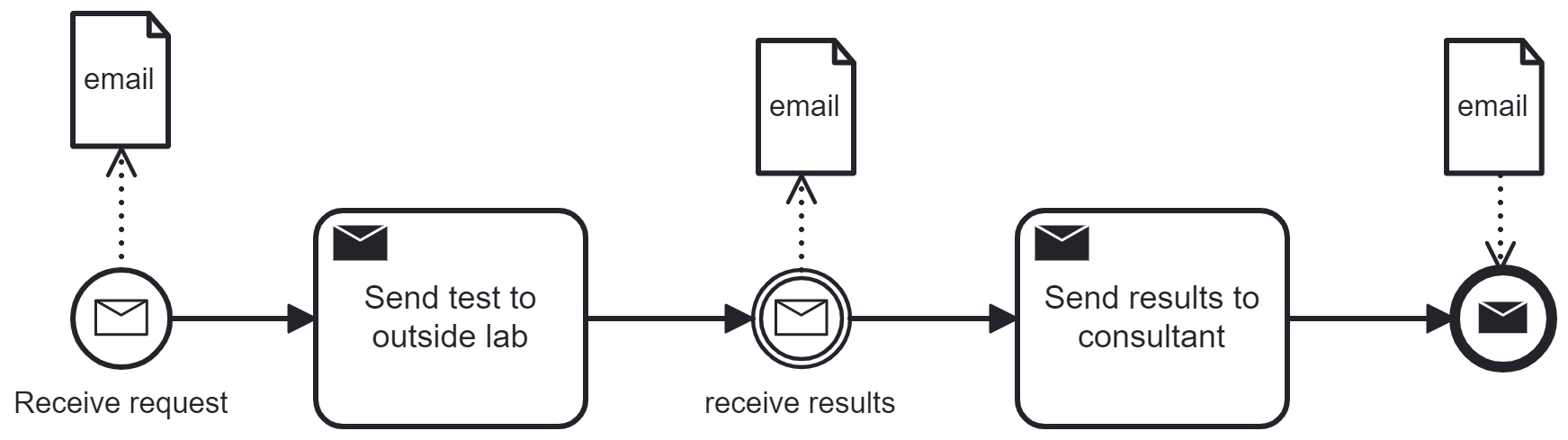

Supplement: Multimedia Appendix 2 [file medinform-v12-e60017-s002.zip › 60017-910936-1-SP.png]

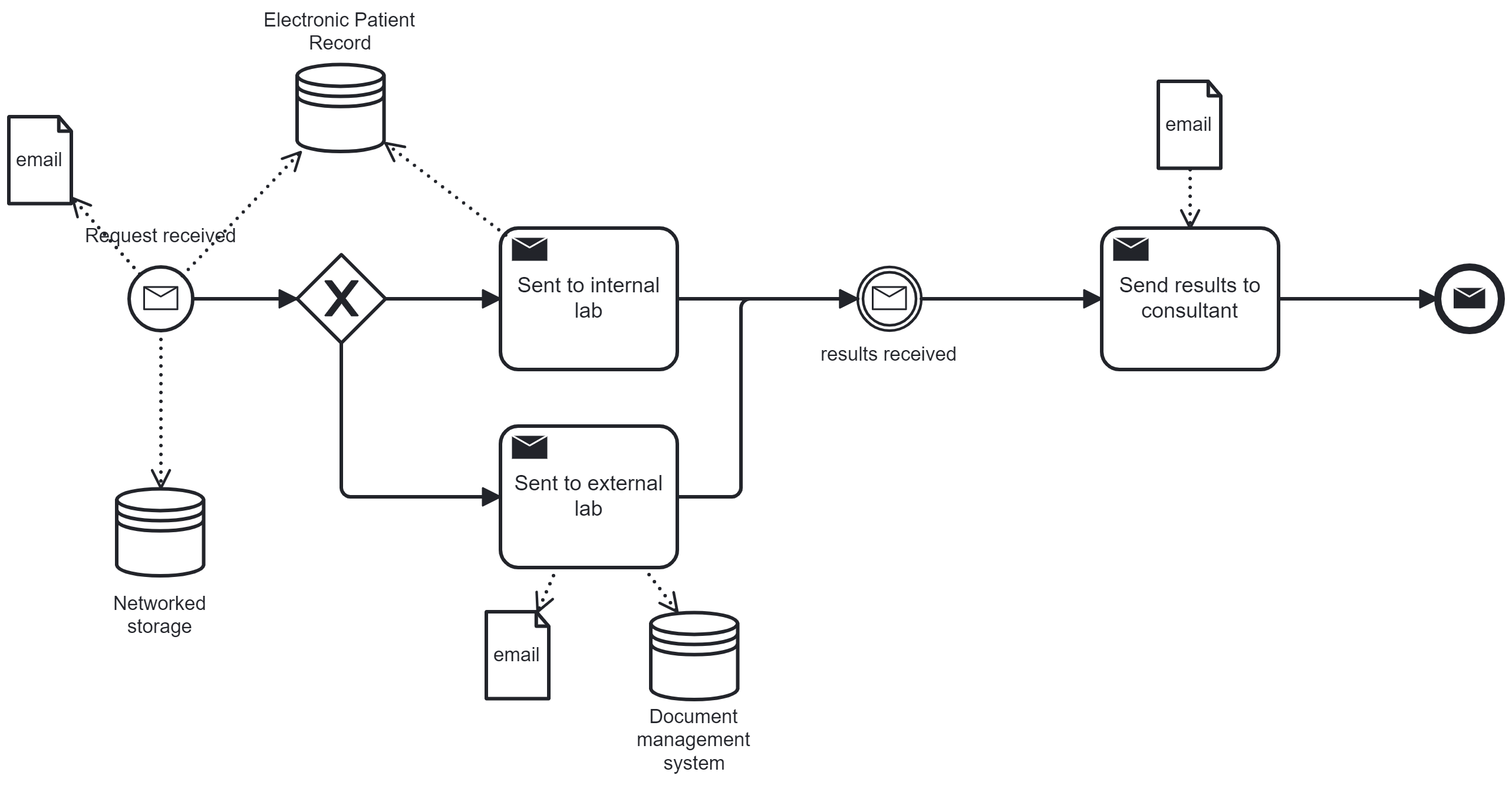

Supplement: Multimedia Appendix 2 [file medinform-v12-e60017-s002.zip › 60017-910937-1-SP.png]

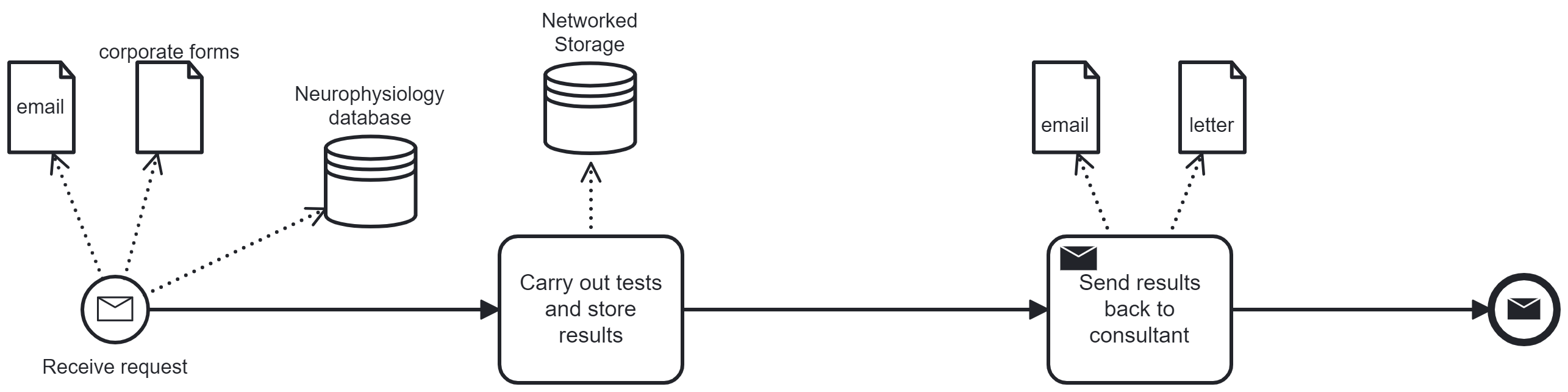

Supplement: Multimedia Appendix 2 [file medinform-v12-e60017-s002.zip › 60017-910941-1-SP.png]

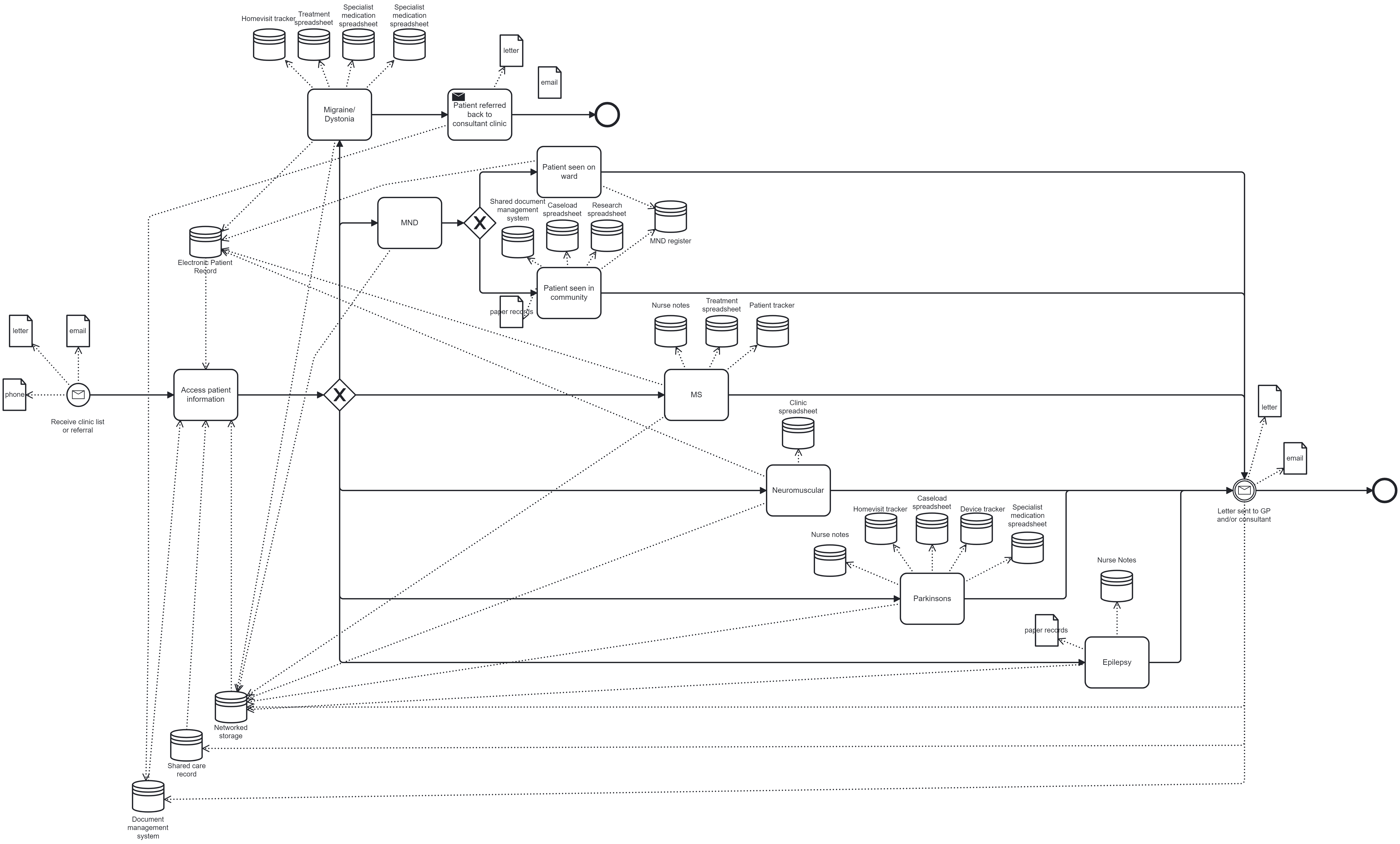

Supplement: Multimedia Appendix 2 [file medinform-v12-e60017-s002.zip › 60017-910946-1-SP.png]
